# Mussel-Inspired Approach to Constructing Dual Network Coated Layered Clay for Enhanced Barrier and Antibacterial Properties of Poly(vinyl alcohol) Nanocomposites

**DOI:** 10.3390/polym12092093

**Published:** 2020-09-15

**Authors:** Long Mao, Jianda Xie, Huiqing Wu, Yuejun Liu

**Affiliations:** 1 Fujian Provincial Key Laboratory of Functional Materials and Applications, Xiamen University of Technology, Xiamen 361024, China; 2013113206@xmut.edu.cn; 2 Key Laboratory of Advanced Packaging Materials and Technology of Hunan Province, Hunan University of Technology, Zhuzhou 412007, China; 3 Key Laboratory of Science & Technology of Eco-Textile, Ministry of Education, College of Chemistry, Chemical Engineering and Biotechnology, Donghua University, Shanghai 201620, China; Huiqingwu@dhu.edu.cn

**Keywords:** layered clay, tannic acid, titanium, poly(vinyl alcohol), barrier properties, active packaging

## Abstract

Inspired by complexation and mussel adhesion of catechol groups in tannic acid (TA), organophilic layered double hydroxides (LDHs@TA-Ti) were synthesized by forming a one-pot assembled TA-titanium (Ti) dual network coating on the surface of layered clay for the first time. LDHs@TA-Ti/poly(vinyl alcohol) (PVA) nanocomposites were prepared by the solution casting method. The results show that TA-Ti(IV) and TiO_2_ coordination compounds are simultaneously formed due to hydrolysis of titanium tetrachloride and complexation of TA in aqueous solution. Upon TA-Ti coatings onto the surface of LDHs, the antibacterial rate of LDHs@TA-Ti is up to 99.98%. Corresponding LDHs@TA-Ti/PVA nanocomposites also show outstanding antibacterial properties. Compared with pure PVA, LDHs@TA-Ti/PVA nanocomposites show a 40.9% increase in tensile strength, a 17.5% increase in elongation at break, a 35.9% decrease in oxygen permeability and a 26.0% decrease in water vapor permeability when adding 1 wt % LDHs@TA-Ti. UV transmittance (at 300 nm) of LDHs@TA-Ti/PVA nanocomposites decrease by 99.4% when the content of LDHs@TA-Ti reaches 3 wt %. These results indicate that PVA matrix incorporated with LDHs@TA-Ti could be used as a potential active packaging material to extend the shelf life of food products.

## 1. Introduction

Over the past few decades, increasingly serious environmental issues and food safety problems have led to the development and application of degradable active packaging materials [1]. Among the widely recognized degradable polymers, poly(vinyl alcohol) (PVA) is a synthetic, versatile and water-soluble polymer used in the packaging industry due to its excellent film forming properties, oxygen barrier properties and chemical resistance [2]. As a kind of non-toxic and non-carcinogenic polymer, PVA is considered to be one of the most promising degradable packaging film materials [3]. However, in the face of various packaging conditions, deficient barrier and antibacterial properties of PVA have limited its wide application in active packaging materials [4]. In order to improve these deficiencies, one of the most effective methods is to prepare nanocomposites with enhanced properties by blending PVA matrix with functional nanoparticles in low volume, while its degradability characteristics remain almost unchanged. In recent years, some studies have been made to improve the individual properties of PVA matrix, such as the water vapor barrier, UV barrier and antibacterial properties, respectively [5]. Nevertheless, there are rare reports on the improvement of the overall performance of the PVA matrix, which are the essential condition for the PVA matrix to be selected as active packaging materials in a practical application [2,6,7]. It should be noted that enhanced barrier and antibacterial properties together could significantly improve the function of active packaging materials (e.g., prolonging the shelf life of food and keeping the flavor of food).

Clay mineral is a kind of natural barrier material because of its impermeable layered structure, which prolongs the tortuous path of gas molecules through the composites [8,9]. Layered double hydroxides (LDHs) are a large class of layered anionic synthetic clay, which have high anion exchange capacity and chemical purity, uniform structure and controllable chemical composition [10,11]. In order to achieve uniform dispersion of LDHs in polymer matrix, diverse organic modification methods were introduced to prepare organophilic LDHs. Xie et al. [3] introduced LDHs intercalated with aliphatic long chain molecules (OLDH) as a reinforcing agent into PVA matrix. When the mass fraction of OLDH was 2%, the tensile strength of OLDH/PVA composites was 66% higher than that of pure PVA. In addition, the water vapor permeability of OLDH/PVA composites was decreased by 24.22% when adding only 0.5 wt % OLDH. Marangoni et al. [12] prepared composite films of PVA and OLDH (intercalated with orange dyes) by the solution casting process. The results indicated that mechanical properties and thermal stability, and capable of absorbing UV radiation of OLDH/PVA composites was significantly increased. Based on the above analysis, it is found that there are few studies on the application of organic modified LDHs in the active packaging based on the PVA matrix, which indicates that the use of modified LDHs to increase the antibacterial and barrier properties of the PVA matrix still has great research value. We hope that the organic modification of LDHs can not only improve the interfacial compatibility and dispersion of LDHs in the PVA matrix, but also give them enhanced antibacterial and other active packaging functions, so as to expand the application of LDHs/PVA nanocomposites in the field of active packaging.

Inspired by the multifunctional adhesion of marine adhesive proteins, surface modification of polyphenols is a powerful method to form adhesive and multifunctional coating (such as polydopamine (PDA) and the tannic acid (TA)–metal ion complex) onto almost any substrate [13,14]. In our previous work, we have reported a facile and green method to prepare organophilic LDHs coated by TA-Fe(III) coordination compounds (LDHs@TA-Fe(III)) [15]. Compared with frequently used PDA coating, the raw material of TA is relatively cheap and easy to obtain, which makes its wide application possible. The results showed that the thermal stability, mechanical and gas barrier properties of LDHs@TA-Fe(III)/Poly(ε-caprolactone) nanocomposite films were markedly improved with the addition of LDHs@TA-Fe(III). The enhancement effect brought by LDHs@TA-Fe(III) is significant owing to enhanced interfacial interactions between layered clay and the matrix. It indicates that mussel-inspired modified LDHs has great research significance in LDHs/polymer nanocomposites.

**Scheme 1:**
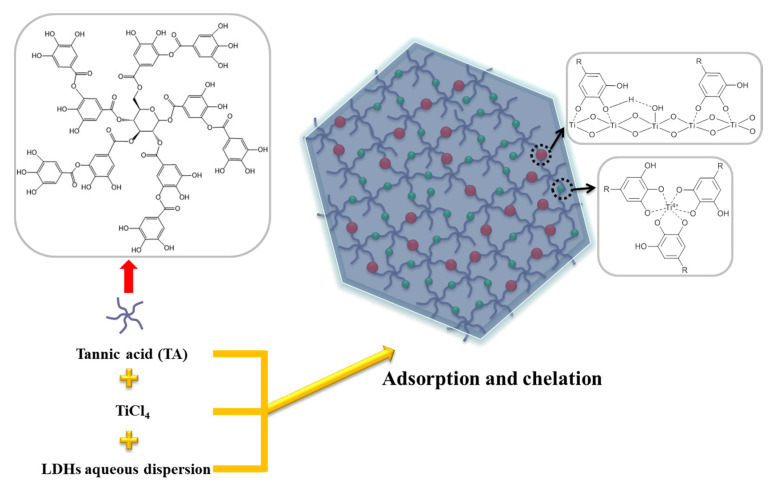
Schematic illustration for the synthesis procedure of layered double hydroxides (LDHs)@TA-Ti via a one-pot assembled TA-Ti dual network coating on the LDHs surface.

In this work, LDHs@TA-Ti was firstly synthesized via a one-pot assembled TA-titanium (Ti) dual network coating on the LDHs surface, to the best of our knowledge (as shown in Scheme 1). In the process of modification, titanium tetrachloride (TiCl_4_) was chosen as the source of titanium ions, because the hydrolysis reaction of TiCl_4_ and chelation reaction of TA were expected to occur spontaneously into aqueous solution. Thus TA-Ti(IV) and TiO_2_ complexes could be simultaneously introduced into the network coating as crosslinking agents, which were quite different from the simple TA–metal ion coordination systems. The functional and organic coating can help to improve the interface interaction and dispersion of LDHs and antibacterial activity of the PVA matrix contributing to excellent comprehensive performance. Additionally, LDHs@TA-Ti/PVA nanocomposites were prepared by the solution casting process. The effect of LDHs@TA-Ti on the thermal, mechanical, barrier and antibacterial properties of LDHs@TA-Ti/PVA nanocomposites was investigated.

## 2. Materials and Methods

### 2.1. Materials and Chemicals

Polyvinyl alcohol (PVA; EXCEVAL™ AQ-4104, Viscosity = 17 mPa·s) was purchased from Kuraray Co., Ltd. (Osaka, Japan). Tannic acid (TA; analytically pure) and titanium tetrachloride (TiCl_4_; analytical pure, the purity ≥ 99.0%) were supplied by Shanghai Aladdin Bio-Chem Technology Co., Ltd. (Shanghai, China). Ni(NO_3_)_2_·6H_2_O, AlCl_3_·6H_2_O and urea were supplied by Xilong Scientific Co., Ltd. (Shantou, China).

### 2.2. Synthesis of NiAl-LDHs

Typically, a mixed aqueous solution was prepared by dissolving Ni(NO_3_)_2_·6H_2_O (0.907 g, 3.12 mmol), AlCl_3_·6H_2_O (0.585 g, 1.56 mmol) and urea (0.380 g, 6.32 mmol) in deionized water. Then the mixed aqueous solution was transferred into a teflon-lined stainless steel autoclave. The autoclave was sealed and heated at 140 °C for 24 h. The NiAl-LDHs slurry was centrifuged, washed and finally dried in a freeze dryer.

### 2.3. Synthesis of LDHs@TA-Ti

NiAl-LDHs (0.1 g) were dispersed in deionized water (200 mL) under ultrasonic treatment for 30 min. Subsequently TA (0.1 g, 0.059 mmol) was added into the NiAl-LDHs dispersion under magnetic stirring for 15 min. Then, TiCl_4_ (2.6 μL, 0.024 mmol) was added into the above dispersion under magnetic stirring for 2 h. In the process of the reaction, the color of the above dispersion gradually changed to reddish brown. LDHs@TA-Ti was obtained by centrifugation and finally dried in a freeze dryer.

### 2.4. Preparation of LDHs@TA-Ti/PVA Nanocomposites

Table 1 lists the composition and naming of LDHs@TA-Ti/PVA nanocomposites. Generally, a certain amount of LDHs@TA-Ti was dispersed in deionized water (14 mL) under ultrasonic treatment for 30 min. Then, PVA was added to LDHs@TA-Ti dispersion at room temperature under magnetic stirring for 15 min. Subsequently, the above dispersion was heated to 95 °C under magnetic stirring for 1 h. In order to remove bubbles, the above dispersion was treated with ultrasonic treatment for 15 min. Finally, the above dispersion was evaporated to obtain homogeneous films in the horizontal polytetrafluoroethylene molds at 50 °C. For comparison, the same processing was used for preparing NiAl-LDHs/PVA nanocomposites (named as LP-1%, NiAl-LDHs loading was 1 wt %).

### 2.5. Characterization

Fourier transform infrared spectroscopy (FT-IR) spectra were recorded in the frequency range of 400–4000 cm^−1^ on a spectrophotometer (ALPHA, Bruker, Billerica, MA, USA). The samples were tested by the attenuated total reflection (ATR) mode.

UV–Vis absorption spectra and light transmittance were recorded by a UV–visible spectrophotometer (SPECORD 210 PLUS, Analytikjena, Jena, Germany) in the wavelength range of 200–1100 nm. The opacity of films was calculated with the following formula [16], opacity = Abs_600_/*t*, where Abs_600_ was the value of absorbance at 600 nm and *t* was the thickness of the film (mm).

X-ray diffraction (XRD) patterns were recorded by a powder diffractometer (X’pert, Panalytical, Almelo, Netherlands), using Cu Kα radiation in the diffraction angle range of 5–80°. 

X-ray photoelectron spectroscopy (XPS) spectra were recorded by an X-ray photoelectron spectrophotometer (K-Alpha, Thermo Fisher Scientific, Waltham, MA, USA) equipped with an Al Kα X-ray source.

Morphological observations were performed with a scanning electron microscope (SEM) (sigma500, Zeiss, Oberkochen, Germany) and transmission electron microscope (TEM; Talos, FEI, Eindhoven, Netherlands), respectively. For the analysis of the fracture surface, the samples were fractured in liquid nitrogen and then coated with gold. Additionally, the elemental distribution and content were analyzed by energy-dispersive X-ray spectrometry (EDS; X-Max^n^, Oxford, Abingdon, UK).

Antibacterial activity against *Escherichia coli* (*E. coli*) was investigated using the shake flask method [17]. Bacterial solution of *E. coli* was cultured by the above method. Powders (mass = 50 mg) and films (diameter = 1 cm), were immersed into falcon tubes containing the above bacterial solution, respectively. After shaking vigorously in a shaking incubator at 37 °C for 24 h, each diluent was spread evenly onto the agar plate. Finally, viable microbial colonies were counted after incubation at 37 °C for 24 h. The antibacterial activity was evaluated by the antibacterial rate calculated with the following formula, the antibacterial rate = [(control value − sample value)/control value] × 100%.

Differential scanning calorimeter (DSC; DSC214, Netzsch, Selb, Germany) was employed for thermal analysis using nitrogen as the protection gas. The samples were measured in the temperature range from room temperature to 230 °C at a heating rate of 20 °C/min, and kept at 230 °C for 5 min before cooling down to eliminate thermal history. Then, the samples were cooled down to −20 °C at a cooling rate of 10 °C/min, and subsequently heated to 230 °C at a heating rate of 10 °C/min. The crystallinity (χ) of PVA was calculated using the following expression, χ = [∆*H*_m_/(∆*H*_0_ × *φ*)] × 100%, where ∆*H*_m_ was the experimental melting enthalpy of the samples, ∆*H*_0_ was the melting enthalpy of 100% crystalline PVA (163 J/g) [18] and *φ* was the mass fraction of PVA in LDHs@TA-Ti/PVA nanocomposites.

A microcontrolled electronic universal testing machine (ETM502B-Ex, Wance, Shenzhen, China) was employed to measure the mechanical properties. The tensile rate was 20 mm/min. Five replicates for each sample were tested and the mean values along with standard deviation of each sample were reported in the results.

The oxygen gas transmission rate (OTR) was measured on an oxygen permeability analyzer (OX-TRAN 2/21, Mocon, Minneapolis, MI, USA). Oxygen permeability tests were conducted at 23 °C on circular films (5 cm^2^) and complies with the ASTM D3985 standard using high purity oxygen gas (>99.999%) at zero relative humidity. Oxygen permeability is equal to the product of OTR and the thickness of the sample. The unit of oxygen permeability is cm^3^·μm·m^−2^·24 h^−1^.

The water vapor transmission rate (WVTR) was tested on the water vapor permeation analyzer (PERMATRAN-W 3/33, Mocon, Minneapolis, MI, USA) at 37.8 °C and 90% relative humidity. The testing complies with the ASTM F1249 standard. Water vapor permeability is equal to the product of WVTR and thickness of sample. The unit of water vapor permeability is g·μm·m^−2^·24 h^−1^.

## 3. Results and Discussion

### 3.1. Chemical Structure of NiAl LDHs and LDHs@TA-Ti

**Figure 1:**
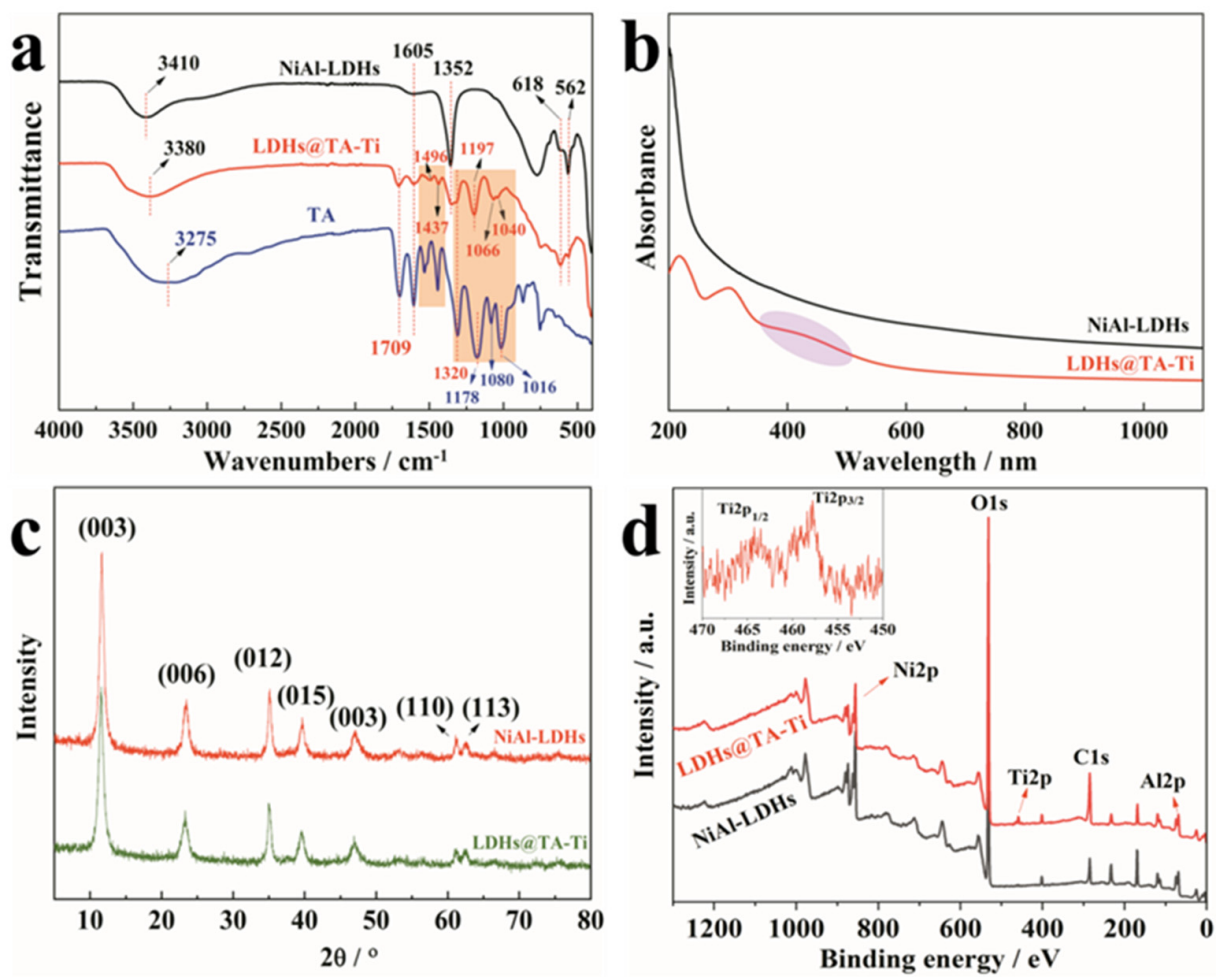
(**a**) FT-IR spectra, (**b**) UV–Vis spectra, (**c**) XRD patterns and (**d**) XPS spectra of NiAl-LDHs and LDHs@TA-Ti.

FT-IR spectra of NiAl-LDHs and LDHs@TA-Ti are shown in Figure 1a. The infrared absorption peak of NiAl-LDHs at 3410 cm^−1^ can be attributed to the characteristic stretching vibration of –OH and the water molecules in the interlayer. Additionally, the infrared absorption peak at 1605 cm^−1^ was attributed to the bending vibrations of the hydrogen bond in water molecules. Affected strongly by phenolic hydroxyl groups in TA, the infrared absorption peak of hydroxyl groups for LDHs@TA-Ti shifted to 3380 cm^−1^. Additionally, the infrared absorption peak at 1352 cm^−1^ for NiAl-LDHs and LDHs@TA-Ti was attributed to the vibrations of CO_3_^2^⁻ in the interlayer [19]. Compared with NiAl-LDHs, LDHs@TA-Ti shows additional peaks at 1709, 1496, 1320 and 1197 cm^−1^, which corresponded to C=O (ester groups), C–O, aromatic C=C and phenolic hydroxyl in TA, respectively [20,21,22]. In addition, the bands recorded below 700 cm^−1^ were attributed to metal-oxygen vibration in NiAl-LDHs [15]. UV–Vis spectra were employed to investigate the characteristic group in LDHs@TA-Ti. As shown in Figure 1b, there was no characteristic absorption peak in NiAl-LDHs. Compared with NiAl-LDHs, a new broad band around 440 nm appeared, which could be attributed to the UV absorption of the Ti(IV)-catechol complex [23]. It is preliminary evidence that TA-Ti coordination compounds were coated on the surface of NiAl-LDHs.

It can be seen that the XRD patterns of NiAl-LDHs and LDHs@TA-Ti both show almost identical characteristics of typical MgAl-LDHs (JCPDS file no. 38-0487), as shown in Figure 1c. XRD patterns both show (003) and (006) crystal plane diffraction peaks of a layered structure and a (110) crystal plane diffraction peak of a lamellar structure [19]. The baseline of these diffraction peaks was low and the peak shape was sharp, which indicates that the layered structure had good regularity. There was no characteristic diffraction peak of TA in the XRD pattern of LDHs@TA-Ti, which was the result of a diffuse and weak diffraction reflection of TA. Additionally, no characteristic reflections of crystalline TiO_2_ could be observed, which indicates that only amorphous TiO_2_ existed in the TA-Ti coordination compounds. From the results of the XPS analysis (Figure 1d), the peak at 459.1 eV attributed to Ti2p was observed in the spectrum of LDHs@TA-Ti. From the high-resolution XPS spectrum of Ti2p (Figure 1d inset), the peaks of Ti2p_1/2_ and Ti2p_3/2_ were observed at 464.2 and 457.8 eV and were assigned to Ti(IV) species, respectively, confirming the presence of TA-Ti coordination compounds on the surface of LDHs [24,25]. Meanwhile, the ratio of C/O for LDHs@TA-Ti (0.52) was higher than that of NiAl-LDHs (~0.21), and was affected by the theoretical value of TA (1.65). Therefore, the XPS results can be effective proof for the introduction of Ti(IV) species and TA on the NiAl-LDHs surface.

### 3.2. Microstructure of NiAl LDHs and LDHs@TA-Ti

**Figure 2:**
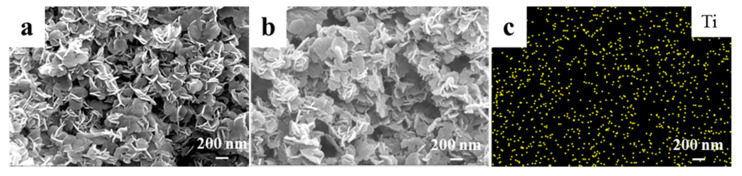
SEM images of (**a**) NiAl-LDHs and (**b**) LDHs@TA-Ti. (**c**) Ti-distribution EDS map-scanning of LDHs@TA-Ti.

The SEM images of NiAl-LDHs and LDHs@TA-Ti are shown in Figure 2. NiAl-LDHs and LDHs@TA-Ti both clearly show a typical layered structure with the particle size of 200 nm and uniform size distribution. Meanwhile, compared with NiAl-LDHs (Figure 2a), the micromorphology of LDHs@TA-Ti was rougher (Figure 2b). Figure 2c demonstrates that Ti element was uniformly distributed on the NiAl-LDHs surface, which reflected the uniform distribution of TA-Ti coordination compounds to some extent.

**Figure 3:**
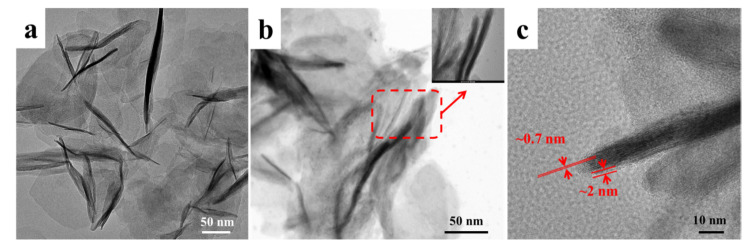
TEM images of (**a**) NiAl-LDHs and (**b**,**c**) LDHs@TA-Ti under different magnifications.

In order to obtain direct evidence of TA-Ti coating on the NiAl-LDHs surface, as shown in Figure 3a,b, the micromorphology of LDHs@TA-Ti is further investigated by TEM. It is revealed that NiAl-LDHs are successfully coated by TA-Ti coordination compounds during the adsorption and chelation process. Estimated from TEM images in Figure 3c, the thickness of TA-Ti coating was 2 nm. Therefore, the lateral thickness of NiAl-LDHs and LDHs@TA-Ti were 10 and 14 nm, respectively. In addition, we also observed that the lattice thickness of NiAl-LDHs was 0.7 nm, which is consistent with the report in the literature [8].

**Figure 4:**
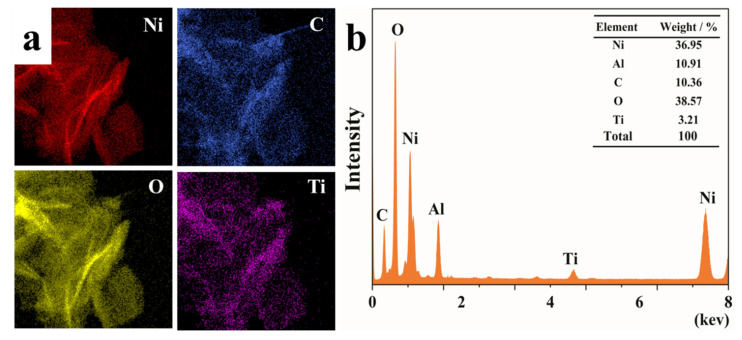
(**a**) EDS map-scanning and (**b**) EDS spectrum of LDHs@TA-Ti.

The chemical composition of LDHs@TA-Ti was analyzed by EDS, as shown in Figure 4. In Figure 4a, EDS map-scanning (scanning area is from Figure 3b) shows the uniform distribution of elements reflected the uniform distribution of the TA-Ti coating. It is revealed that the original LDHs were well coated by TA-Ti coordination compounds. In Figure 4b, the large peaks of nickel and aluminum in the EDS spectrum indicates the basic components of NiAl-LDHs. It is worth noting that the small peak of titanium indicates the existence of TA-Ti(IV) and TiO_2_ coordination compounds [26]. 

### 3.3. Antibacterial Properties of LDHs@TA-Ti and LDHs@TA-Ti/PVA Nanocomposites

**Figure 5:**
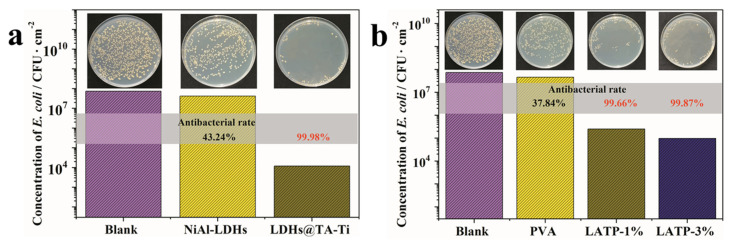
Antibacterial properties of (**a**) NiAl-LDHs and (**b**) LDHs@TA-Ti/PVA nanocomposites.

Antibacterial properties of LDHs@TA-Ti and LDHs@TA-Ti/PVA nanocomposites are shown in Figure 5. In Figure 5a, the antibacterial rate of NiAl-LDHs only reached 43.24%. After TA-Ti coordination compounds were coated on the NiAl-LDHs surface, the antibacterial rate of LDHs@TA-Ti reached 99.98%. The excellent antibacterial activity of LDHs@TA-Ti is the result of the synergistic effect from phenolic hydroxyl groups and photocatalytic titanium dioxide [23,27]. Therefore, LDHs@TA-Ti can be a multifunctional additive to effectively inhibit the growth of bacteria and solve the problem caused by the direct addition of a single antibacterial agent. In general, the antibacterial properties of nanoparticles are closely related to that of the nanocomposites prepared by them. In Figure 5b, the antibacterial rate of LATP-1% and LATP-3% both exceeded 99.5%. Therefore, with the increase of LDHs@TA-Ti content, the antibacterial properties of LDHs@TA-Ti/PVA nanocomposites become more and more excellent. 

### 3.4. Chemical Structure of LDHs@TA-Ti/PVA Nanocomposites

**Figure 6:**
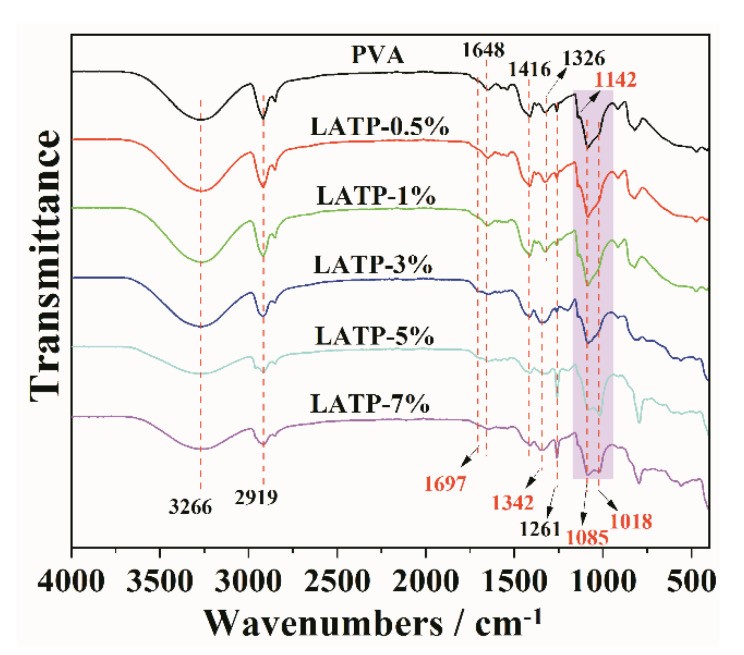
FT-IR spectra of LDHs@TA-Ti/PVA nanocomposites.

In order to investigate the changes of the internal chemical structure for LDHs@TA-Ti/PVA nanocomposites, FT-IR analysis was performed as shown in Figure 6. In the case of the pure PVA spectrum, the characteristic absorption peaks at 3266, 1326 and 1085 cm^−1^ were caused by the O–H stretching vibration, CH–OH bending vibration and C–O stretching vibration, respectively. Additionally, the absorption peaks at 2919 and 1416 cm^−1^ were attributed to the asymmetric stretching vibration and bending vibration of C–H groups respectively [16,28]. With the addition of LDHs@TA-Ti, the weak absorption peak of ester groups in TA appeared near 1697 cm^−1^. The bending vibration peak of CH–OH groups at 1326 cm^−1^ shifted to 1342 cm^-1^ due to the influence of carbonate and phenolic hydroxyl in LDHs@TA-Ti [23]. In the range of 1200–950 cm^−1^, with the increase of LDHs@TA-Ti content, the stretching vibration peaks of C–O groups were obviously widened, which indicates that there was a strong interaction between LDHs@TA-Ti and PVA. 

Further, based on previous research [29,30,31], the C–O stretching vibration peaks of 1142 and 1085 cm^−1^ represent the “free” C–O stretching vibration peaks and the C–O stretching vibration peaks caused by the hydrogen bonding of C–O and O–H functional groups, respectively. With the addition of LDHs@TA-Ti to a certain extent, the absorption peak gradually strengthened at 1018 cm^−1^, which was caused by C–O groups after the formation of hydrogen bond between C–O and phenolic hydroxyl in TA.

### 3.5. Thermal and Crystalline Properties of LDHs@TA-Ti/PVA Nanocomposites

**Figure 7:**
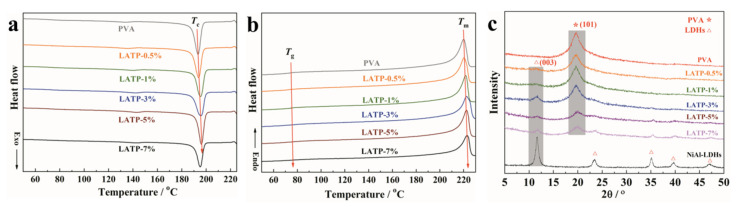
(**a**) Differential scanning calorimeter (DSC) cooling curves, (**b**) DSC second heating curves and (**c**) XRD patterns of LDHs@TA-Ti/PVA nanocomposites.

In order to investigate the effect of LDHs@TA-Ti on the thermal properties of the PVA matrix, DSC analysis was carried out on LDHs@TA-Ti/PVA nanocomposites, as shown in Figure 7a,b. Table 2 presents the corresponding data of thermal parameters, which includes glass transition (*T*_g_), crystallization temperature (*T*_c_), melting temperature (*T*_m_), melting enthalpy (Δ*H*_m_) and crystallinity (*χ*). The *T*_g_ of PVA was 74.8 °C, while that of the LDHs@TA-Ti/PVA nanocomposites increased slightly. This can be due to the confinement effects and strong interaction between polymer and filler particles, which leads to restricted segmental mobility of PVA chains in the vicinity of LDHs@TA-Ti [18,32]. The increase of *T*_c_ is helpful to the formation of a more perfect crystal structure, which leads to an increase in *T*_m_ eventually. Different from the crystallization characteristics of the general LDHs/polymer nanocomposites, *χ* of LDHs@TA-Ti/PVA nanocomposites shows a decreasing trend with the addition of LDHs@TA-Ti. The crystal quantity and regularity of PVA matrix is broken as a consequence of strong interaction between polymer and filler particles [33]. Therefore, *χ* of LDHs@TA-Ti/PVA nanocomposites decreased from 30.33% to 25.12%.

The crystalline properties of LDHs@TA-Ti/PVA nanocomposites were also investigated by XRD in Figure 7c. LDHs@TA-Ti/PVA nanocomposites exhibited a strong diffraction peak at 2θ = 19.6°, corresponding to the (101) crystal face of the PVA matrix [12,34]. With the addition of LDHs@TA-Ti, the diffraction peak intensity of LDHs@TA-Ti/PVA nanocomposites decreased gradually. This further confirms the results of the above thermal analysis. Moreover, all the XRD patterns show the (003) diffraction peak at 2θ = 11.6°. The diffraction peak intensity increased with the addition of LDHs@TA-Ti, which indicates that LDHs@TA-Ti hindered the crystallization behavior of PVA chains.

### 3.6. Mechanical Properties and the Microstructure of LDHs@TA-Ti/PVA Nanocomposites

**Figure 8:**
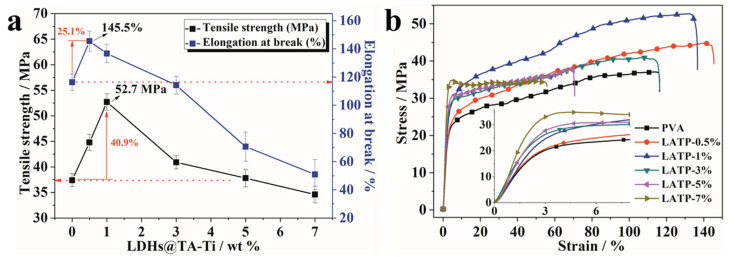
(**a**) Mechanical properties and (**b**) stress–strain curves of LDHs@TA-Ti/PVA nanocomposites.

Figure 8 shows the mechanical properties and stress–strain curves of LDHs@TA-Ti/PVA nanocomposites. Compared with pure PVA, the tensile strength and elongation at the break of LATP-1% increased by 40.9% and 17.5%, respectively. Additionally, the tensile strength of LDHs@TA-Ti/PVA nanocomposites continued to increase when the addition of LDHs@TA-Ti was less than 1 wt %. The reason for the increase of tensile strength was due to the strong interfacial interaction and adhesion between the PVA matrix and LDHs@TA-Ti, which hindered the movement of the chain segments and restricted the deformation ability of the chain segments, finally resulting in the enhancement effect, which improved the resistance to the external force [35]. When the addition of LDHs@TA-Ti exceeded 1 wt %, the tensile strength of LDHs@TA-Ti/PVA nanocomposites began to decrease gradually. The elongation at the break of LDHs@TA-Ti/PVA nanocomposites reached the maximum value when the mass fraction of LDHs reached 0.5%, and then decreased continuously. Nonetheless, the tensile strength and elongation at the break of LDHs@TA-Ti/PVA nanocomposites were both higher than those of pure PVA when the LDHs@TA-Ti content reached 3 wt %. As a general rule, the strength of composites depends on the size and distribution of defects caused by fillers, which determines the loading degree of composites before failure [36]. Therefore, it is noteworthy that the PVA matrix treated with an appropriate amount of LDHs@TA-Ti had better mechanical properties than pure PVA.

As shown in Figure 8b, all samples had no obvious boundary in the forced high-elastic deformation and strain hardening stage. This is consistent with the tensile behavior of PVA reported in the literature [12]. Different from the tensile strength, the yield strength of all the LDHs@TA-Ti/PVA nanocomposites was higher than that of pure PVA. This is related to the enhancement effect caused by the addition of LDHs@TA-Ti. The strain of LDHs@TA-Ti/PVA nanocomposites was larger than that of pure PVA when LDHs@TA-Ti content was less than 3 wt %. The larger deformation range before the fracture indicates the stronger interfacial interactions between LDHs@TA-Ti and the PVA matrix. This is a general trend observed in nanocomposite systems because of their higher flexibility. Moreover, we can get the elastic modulus by linear fitting of the elastic deformation stage in the strain–stress curves. PVA presents an elastic modulus value of 852 MPa. The elastic modulus values of LDHs@TA-Ti/PVA nanocomposites were 945, 1026, 1147, 1220 and 1518 MPa for 0.5%, 1%, 3%, 5% and 7% of added filler, respectively. With the increase of LDHs@TA-Ti content, the elastic modulus of LDHs@TA-Ti/PVA nanocomposites increased gradually. It is worth noting that the elastic modulus of typical LDHs was 2470 MPa [15]. Therefore, a significant increase in elastic modulus could be attributed to the high modulus of the filler and strong interface interaction.

**Figure 9:**
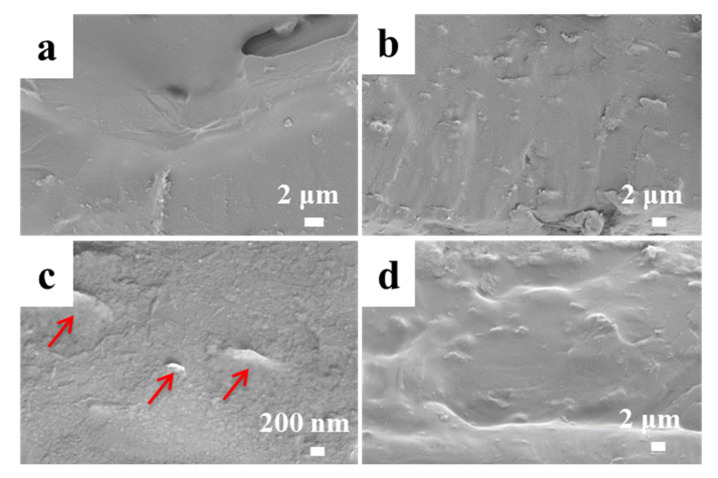
SEM images of the fracture surface for PVA and LDHs@TA-Ti/PVA nanocomposites ((**a**), PVA; (**b**,**c**), LATP-1%; (**d**), LATP-5%).

As shown in Figure 9a, the overall fracture surface of pure PLA was rough, which indicates plastic deformation. Compared with pure PVA, the fracture surface (Figure 9b) of LATP-1% was much more rough. A large number of the lamellar stretched surface appeared orderly on the fracture surface of LATP-1%, which indicates that LDHs@TA-Ti was uniformly dispersed in the PVA matrix. In Figure 9c, LDHs@TA-Ti tightly combined with the PVA matrix without a visible interface gap. Additionally, there was no visible agglomeration of LDHs@TA-Ti. Generally, relatively good dispersion of nanoparticles can be achieved in the nanocomposites with low nanoparticle content. In Figure 9d, apparently, LDHs@TA-Ti shows a heterogeneous stretched surface on the fracture surface of LATP-5%, which indicates that the dispersibility of LDHs@TA-Ti got worse. To some extent, this could explain the reasons for the change of mechanical properties with the LDHs@TA-Ti contents increase. Meanwhile, the better the interfacial bonding, the fewer the interfacial defects, which is also closely related to the better gas barrier properties of materials.

### 3.7. UV–Vis Barrier Properties and Transparency of LDHs@TA-Ti/PVA Nanocomposites

**Figure 10:**
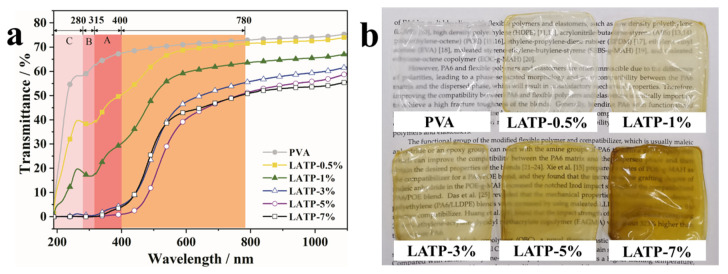
(**a**) UV–Vis light transmittance and (**b**) digital photographs of LDHs@TA-Ti/PVA nanocomposites.

Protecting food from the effect of UV–Vis radiation is one of the important characteristics of the active packaging material. The reason is that it will affect product performance and consumer acceptance [37]. The UV–Vis transmittance of LDHs@TA-Ti/PVA nanocomposites is shown in Figure 10a. UV barrier property is an important parameter for food packaging applications, which can minimize the lipid oxidation induced by UV radiation, preserve the sensory characteristics of packaged food, avoid the loss of nutrients, discoloration and off-odor, so as to prolong the shelf life of food [38]. In the UV range (200–400 nm), it is obvious that LDHs@TA-Ti/PVA nanocomposites incorporating with the addition of LDHs@TA-Ti show much lower light transmission levels. Taking the UV wavelength at 300 nm as an example, when the LDHs@TA-Ti content reached 1 wt %, UV transmittance of LDHs@TA-Ti/PVA nanocomposites decreased by 75.8% (compared with pure PVA). Additionally, the UV transmittance of LATP-3% further decreased by 99.4%. In the visible range (400-780 nm), light transmittance was also reduced at increasing LDHs@TA-Ti content. However, the decreasing range of light transmittance was not as large as that of the UV region. Taking the wavelength at 600 nm as an example, even if the LDHs@TA-Ti content reached 5 wt %, the light transmittance of LDHs@TA-Ti/PVA nanocomposites decreased by only 33.5%. Therefore, it indicates that the barrier effect of LDHs@TA-Ti on UV light was obviously stronger than that of visible light. In addition, by comparing the light transmittance of LP-1% and LATP-1%, it can be found that the UV–Vis barrier ability of LDHs@TA-Ti/PVA nanocomposites mainly came from TA-Ti coordination compounds.

The opacity of pure PVA and LDHs@TA-Ti/PVA nanocomposites was 2.46, 3.07, 3.75, 5.42, 6.58 and 6.95, respectively. With the addition of LDHs@TA-Ti, the transparency of LDHs@TA-Ti/PVA nanocomposites distinctly decreased. In particular, when the LDHs@TA-Ti content exceeded 1 wt *%*, the opacity of LDHs@TA-Ti/PVA nanocomposites reached 5.42, which revealed that it could not provide a clear view of the food content and its condition. Compared with the opacity (2.72) of LP-1%, the opacity of LATP-1% increased significantly, which indicates that the TA-Ti coordination compounds further reduced the transparency of LDHs@TA-Ti/PVA nanocomposites. Therefore, the opacity value of LATP-0.5% and LATP-1% at 600 nm was lower than 5 [16], so they could be considered as transparent films.

Digital photographs of LDHs@TA-Ti/PVA nanocomposites are shown in Figure 10b. With the addition of LDHs@TA-Ti, the color of LDHs@TA-Ti/PVA nanocomposites is getting darker and darker. Although these films have a certain degree of curling, which affects the visual transparency. The visual transparency is similar to the light transmittance in the above results.

### 3.8. Gas Barrier Properties of LDHs@TA-Ti/PVA Nanocomposites

**Figure 11:**
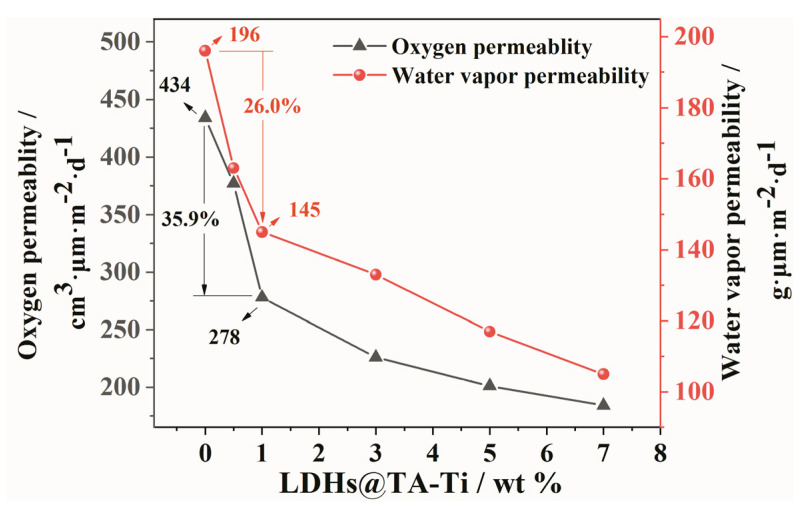
Oxygen permeability and water vapor permeability of LDHs@TA-Ti/PVA nanocomposites.

Oxygen permeability and water vapor permeability are important properties of active packaging materials, which are closely related to the internal and external environment conditions of food packaging. Gas barrier properties of LDHs@TA-Ti/PVA nanocomposites are shown in Figure 11. Compared with pure PVA, oxygen permeability and water vapor permeability of LATP-1% decreased by 35.9% and 26.0%, respectively, which were mainly due to the barrier properties of LDHs@TA-Ti and the interface interaction between LDHs@TA-Ti and PVA. With the increase of LDHs@TA-Ti content, oxygen permeability and water vapor permeability of LDHs@TA-Ti/PVA nanocomposites decreased gradually. However, the barrier efficiency will be greatly reduced by adding a high amount of nanoparticles. When the LDHs@TA-Ti content reached 7%, oxygen permeability and water vapor permeability of LDHs@TA-Ti/PVA nanocomposites were both the lowest. Compared with pure PVA, oxygen permeability and water vapor permeability of LATP-7% only decreased by 57.6% and 46.4%, respectively. This may be due to the local agglomeration of LDHs@TA-Ti in the PVA matrix, which weakens the interface interaction between LDHs@TA-Ti and PVA matrix and forms large-scale holes. From the above analysis, it could be concluded that the appropriate LDHs@TA-Ti content could show good barrier efficiency in the LDHs@TA-Ti/PVA nanocomposites and reduce the negative effects for a high amount of nanoparticles.

In addition, the barrier effect of LDHs@TA-Ti on oxygen is significantly greater than that on water vapor. This is because both PVA and LDHs@TA-Ti have enhanced interaction with polar water molecules. Water absorption and swelling can promote the adsorption and permeation of water particles. Nevertheless, LDHs@TA-Ti can effectively prolong the permeation path of water vapor. Therefore, the interaction of the two mechanisms will eventually reduce the resistance of water vapor to a certain extent.

## 4. Conclusions

In this study, LDHs@TA-Ti was firstly synthesized via a one-pot assembled TA-Ti dual network coating on the of LDHs surface. Additionally, LDHs@TA-Ti/PVA nanocomposites were prepared by the solution casting process to obtain uniform thin films. TA-Ti coordination compounds were coated. The results show that TA-Ti(IV) and TiO_2_ coordination compounds with a thickness of 2 nm were simultaneously compact and uniform on the surface of LDHs due to hydrolysis of titanium tetrachloride and the complexation of TA in aqueous solution. Upon TA-Ti coating onto the LDHs surface, the antibacterial rate of LDHs@TA-Ti was up to 99.98%, as a result of the synergistic effect from the phenolic hydroxyl groups and photocatalytic TiO_2_. Additionally, LDHs@TA-Ti/PVA nanocomposites also show outstanding antibacterial properties. Compared with pure PVA, LDHs@TA-Ti/PVA nanocomposites show a 40.9% increase in tensile strength, a 17.5% increase in elongation at break, a 35.9% decrease in oxygen permeability and a decrease of 26.0% in water vapor permeability when adding 1 wt % LDHs@TA-Ti. Excessive LDHs@TA-Ti content gave rise to a reduction in the mechanical properties and gas barrier efficiency. UV transmittance (at 300 nm) of LDHs@TA-Ti/PVA nanocomposites decreased by 99.4% when LDHs@TA-Ti content reached 3 wt %. Therefore, LDHs@TA-Ti/PVA nanocomposites show a great application potential in the active packaging application due to their mechanical, barrier and antibacterial properties.

## Figures and Tables

**Table 1:** The composition and naming of LDHs@TA-T*i*/PVA nanocomposites.

Sample	PVA/g	LDHs@TA-Ti/g	LDHs@TA-Ti/wt %
PVA	0.7	0	0
LATP-0.5%	0.7	0.0035	0.5
LATP-1%	0.7	0.0071	1
LATP-3%	0.7	0.0216	3
LATP-5%	0.7	0.0368	5
LATP-7%	0.7	0.0527	7

**Table 2:** The related results of LDHs@TA-Ti/PVA nanocomposites according to the thermal analysis.

Sample	*T*_g_/^o^C	*T*_c_/^o^C	*T*_m_/^o^C	Δ*H*_m_/J·g^−1^	χ/%
PLA	74.8	193.3	220.0	49.44	30.33
LATP-0.5%	74.9	193.8	220.3	44.76	27.60
LATP-1%	74.9	195.5	221.7	42.77	26.50
LATP-3%	75.3	195.7	221.5	41.15	26.03
LATP-5%	75.3	196.1	222.4	40.53	26.17
LATP-7%	76.3	195.2	222.9	38.08	25.12

## References

[1] Zeid A., Karabagias I.K., Nassif M., Kontominas M.G. (2019). Preparation and evaluation of antioxidant packaging films made of polylactic acid containing thyme, rosemary, and oregano essential oils. J. Food Process. Pres..

[2] Chen C., Tang Z., Ma Y., Qiu W., Yang F., Mei J., Xie J. (2018). Physicochemical, microstructural, antioxidant and antimicrobial properties of active packaging films based on poly(vinyl alcohol)/clay nanocomposite incorporated with tea polyphenols. Prog. Org. Coat..

[3] Xie J., Zhang K., Wang Z., Zhao Q., Yang Y., Zhang Y., Ai S., Xu J. (2017). Biodegradable poly(vinyl alcohol)-based nanocomposite film reinforced with organophilic layered double hydroxides with potential packaging application. Iran. Polym. J..

[4] Zhou K., Gui Z., Hu Y. (2017). Facile synthesis of LDH nanoplates as reinforcing agents in PVA nanocomposites. Polym. Adv. Technol..

[5] Gaaz T., Sulong A., Akhtar M., Kadhum A., Mohamad A., Al-Amiery A. (2015). Properties and Applications of Polyvinyl Alcohol, Halloysite Nanotubes and Their Nanocomposites. Molecules.

[6] İşçi Y., İşçi S. (2017). Comparison of the clay minerals type on the properties of reinforced-PVA nanocomposites. Polym. Compos..

[7] Huang J., Limqueco J., Chieng Y.Y., Li X., Zhou W. (2017). Performance evaluation of a novel food packaging material based on clay/polyvinyl alcohol nanocomposite. Innov. Food Sci. Emerg..

[8] Zhang Y., Liu Q., Zhang S., Zhang Y., Cheng H. (2015). Gas barrier properties and mechanism of kaolin/styrene–butadiene rubber nanocomposites. Appl. Clay Sci..

[9] Choudalakis G., Gotsis A.D. (2009). Permeability of polymer/clay nanocomposites: A review. Eur. Polym. J..

[10] Tammaro L., Costantino U., Bolognese A., Sammartino G., Marenzi G., Calignano A., Tetè S., Mastrangelo F., Califano L., Vittoria V. (2007). Nanohybrids for controlled antibiotic release in topical applications. Int. J. Antimicrob. Agents.

[11] Pucciariello R., Tammaro L., Villani V., Vittoria V. (2007). New nanohybrids of poly(ε-caprolactone) and a modified Mg/Al hydrotalcite: Mechanical and thermal properties. J. Polym. Sci. Pol. Phys..

[12] Marangoni R., Gardolinski J.E.F.D., Mikowski A., Wypych F. (2011). PVA nanocomposites reinforced with Zn_2_Al LDHs, intercalated with orange dyes. J. Solid State Electr..

[13] Wang C., Han H., Jiang W., Ding X., Li Q., Wang Y. (2017). Immobilization of Thermostable Lipase QLM on Core-Shell Structured Polydopamine-Coated Fe_3_O_4_ Nanoparticles. Catalysts.

[14] Nam H.J., Park E.B., Jung D. (2016). Bioinspired polydopamine-layered double hydroxide nanocomposites: Controlled synthesis and multifunctional performance. RSC Adv..

[15] Mao L., Wu H., Liu Y., Yao J., Bai Y. (2018). Enhanced mechanical and gas barrier properties of poly(ε-caprolactone) nanocomposites filled with tannic acid-Fe(III) functionalized high aspect ratio layered double hydroxides. Mater. Chem. Phys..

[16] Chen C., Xie J., Yang F., Zhang H., Xu Z., Liu J., Chen Y. (2018). Development of moisture-absorbing and antioxidant active packaging film based on poly(vinyl alcohol) incorporated with green tea extract and its effect on the quality of dried eel. J. Food Process. Pres..

[17] Kim H.J., Kim D., Yoon H., Choi Y., Yoon J., Lee J. (2015). Polyphenol/FeIII complex coated membranes having multifunctional properties prepared by a one-step fast assembly. Adv. Mater. Interfaces.

[18] Ramaraj B., Nayak S.K., Yoon K.R. (2010). Poly(vinyl alcohol) and layered double hydroxide composites: Thermal and mechanical properties. J. Appl. Polym. Sci..

[19] Mao L., Liu Y., Wu H., Chen J., Yao J. (2017). Poly(ε-caprolactone) filled with polydopamine-coated high aspect ratio layered double hydroxide: Simultaneous enhancement of mechanical and barrier properties. Appl. Clay Sci..

[20] Fan L., Ma Y., Su Y., Zhang R., Liu Y., Zhang Q., Jiang Z. (2015). Green coating by coordination of tannic acid and iron ions for antioxidant nanofiltration membranes. RSC Adv..

[21] Song Q., Zhao W., Yin H., Lian H. (2015). Facile synthesis of FeIII–tannic acid film-functionalized magnetic silica microspheres for the enrichment of low-abundance peptides and proteins for MALDI-TOF MS analysis. RSC Adv..

[22] Kim S., Kwak S., Lee S., Cho W.K., Lee J.K., Kang S.M. (2015). One-step functionalization of zwitterionic poly[(3-(methacryloylamino)propyl)dimethyl(3-sulfopropyl)ammonium hydroxide] surfaces by metal–polyphenol coating. Chem. Commun..

[23] Wu H., Xie J., Mao L. (2020). One-pot assembly tannic acid-titanium dual network coating for low-pressure nanofiltration membranes. Sep. Purif. Technol..

[24] Zhao X., Jia N., Cheng L., Liu L., Gao C. (2018). Dopamine-induced biomimetic mineralization for in situ developing antifouling hybrid membrane. J. Membr. Sci..

[25] Rahim M.A., Björnmalm M., Suma T., Faria M., Ju Y., Kempe K., Müllner M., Ejima H., Stickland A.D., Caruso F. (2016). Metal-Phenolic Supramolecular Gelation. Angew. Chem. Int. Ed..

[26] Kim H.J., Im S., Kim J.C., Hong W.G., Shin K., Jeong H.Y., Hong Y.J. (2017). Phytic Acid Doped Polyaniline Nanofibers for Enhanced Aqueous Copper(II) Adsorption Capability. ACS Sustain. Chem. Eng..

[27] Mao L., Liu J.Y., Zheng S.J., Wu H.Q., Bai Y.K. (2019). Mussel-inspired nano-silver loaded layered double hydroxides embedded into a biodegradable polymer matrix for enhanced mechanical and gas barrier properties. RSC Adv..

[28] Chen C., Xu Z., Ma Y., Liu J., Zhang Q., Tang Z., Fu K., Yang F., Xie J. (2018). Properties, vapour-phase antimicrobial and antioxidant activities of active poly(vinyl alcohol) packaging films incorporated with clove oil. Food Control.

[29] Zhao L., Yang D., Dong M., Xu T., Jin Y., Xu S., Zhang F., Evans D.G., Jiang X. (2010). Fabrication and Wettability of Colloidal Layered Double Hydroxide-Containing PVA Electrospun Nanofibrous Mats. Ind. Eng. Chem. Res..

[30] He Y., Zhu B., Inoue Y. (2004). Hydrogen bonds in polymer blends. Prog. Polym. Sci..

[31] Ping Z.H., Nguyen Q.T., Chen S.M., Zhou J.Q., Ding Y.D. (2001). States of water in different hydrophilic polymers—DSC and FTIR studies. Polymer.

[32] Zhang J., Liu T., Liu Z., Wang Q. (2019). Facile fabrication of tough photocrosslinked polyvinyl alcohol hydrogels with cellulose nanofibrils reinforcement. Polymer.

[33] Liu M., Guo B., Du M., Jia D. (2007). Drying induced aggregation of halloysite nanotubes in polyvinyl alcohol/halloysite nanotubes solution and its effect on properties of composite film. Appl. Phys. A Mater..

[34] Huang S., Cen X., Zhu H., Yang Z., Yang Y., Tjiu W.W., Liu T. (2011). Facile preparation of poly(vinyl alcohol) nanocomposites with pristine layered double hydroxides. Mater. Chem. Phys..

[35] Patwa R., Kumar A., Katiyar V. (2018). Effect of silk nano-disc dispersion on mechanical, thermal, and barrier properties of poly(lactic acid) based bionanocomposites. J. Appl. Polym. Sci..

[36] Moyo L., Makhado E., Sinha Ray S. (2014). Anomalous impact strength for layered double hydroxide-palmitate/poly(ε-caprolactone) nanocomposites. J. Appl. Polym. Sci..

[37] Hajji S., Chaker A., Jridi M., Maalej H., Jellouli K., Boufi S., Nasri M. (2016). Structural analysis, and antioxidant and antibacterial properties of chitosan-poly (vinyl alcohol) biodegradable films. Environ. Sci. Pollut. Res..

[38] Wu J., Sun X., Guo X., Ge S., Zhang Q. (2017). Physicochemical properties, antimicrobial activity and oil release of fish gelatin films incorporated with cinnamon essential oil. Aquac. Fish..

